# Exploring the Moderation Effects of Race on the Relationship Among Sex Hormones, Biomarkers, and Psychological Symptoms in Female Older Adults

**DOI:** 10.1177/10547738251344980

**Published:** 2025-06-19

**Authors:** Se Hee Min, Maxim Topaz, Chiyoung Lee, Rebecca Schnall

**Affiliations:** 1 University of Pennsylvania School of Nursing, Philadelphia, PA, USA; 2 Columbia University School of Nursing, New York, NY, USA; 3 University of Arizona College of Nursing, Tucson, AZ, USA

**Keywords:** aging, biomarker, cognition, methodology, older adults

## Abstract

With aging, female older adults experience biochemical changes such as drop in their sex hormones and biomarkers and often encounter stress, which can be manifested in psychological symptoms. Previous literature has confirmed that racial/ethnic differences exist in the interactive relationship between sex hormones, biomarkers, and psychological symptoms. Yet, the racial/ethnic differences in their interactive relationship have not yet been examined. This is a secondary data analysis using the cross-sectional data of Wave II (2010–2011) from the National Social Life, Health, and Aging Project (NSHAP), and included 1,228 female older adults without moderate to severe cognitive impairment. Moderated network analysis was conducted with race as a moderator to examine the interactive relationship among sex hormones, biomarkers, and psychological symptoms and to compare the differences between the White and non-White group. The White group had a more positive relationship between total hemoglobin and cognition (edge weight = 0.18; moderated edge weight = 0.22). The non-White group had a positive relationship between progesterone and anxiety (edge weight = 0.05; moderated edge weight = 0.04) and between estradiol and cognition (edge weight = 0.03; moderated edge weight = 0.03), both of which were not present in the White group. We found a small moderated effect of race, and the strength of relationship among sex hormones, biomarkers, and psychological symptoms was different between the White and non-White group. Our study offers important preliminary findings to understand the potential racial/ethnic disparities that exist among sex hormones, biomarkers, and psychological symptoms in female older adults and the need to take an interactive approach.

## Introduction

With aging, older adults experience biopsychosocial changes such as emerging health concerns, biological changes, changes in social networks, retirement from long-held job positions, and deaths of close friend or family members (Aydın et al., 2020). Older adults often encounter stress related to these biopsychosocial changes during this life stage, which can be manifested in psychological symptoms (Thapa et al., 2020). These psychological symptoms include anxiety, depression, loneliness, and cognitive impairment (Korten et al., 2017; Santini et al., 2016). A significant gender difference exists in psychological symptoms, with female older adults more likely to suffer from worse general mental health and thus report more severe depressive and anxiety symptoms (Sialino et al., 2021). In addition, while female older adults have a greater cognitive reserve, they experience faster cognitive decline than males (Levine et al., 2021). Nonetheless, these psychological symptoms are often underdiagnosed and undertreated in older adults (Allan et al., 2014). This may be due to older adults’ perception that their psychological symptoms are a part of normal process of aging and the tendency to underreport their symptoms, or the different clinical presentation of psychological symptoms in older adults that challenge clinicians to accurately diagnose and treat in clinical settings (Allan et al., 2014; Garrido et al., 2011).

Female older adults experience biochemical changes associated with aging and post-menopause that leads to drop in their estrogen, progesterone, and hemoglobin level (Al-Azzawi & Palacios, 2009). Estradiol (E2), a form of estrogen made mainly by the ovaries to mature and maintain the female reproductive system, declines rapidly over the menopausal transition (Russell et al., 2019). Progesterone, another type of female sex hormone produced by the corpus luteum to regulate menstruation and maintain pregnancy, also begins to decline with aging (Henderson, 2018). Such decline of sex hormones has been associated with changes in brain that leads to adverse effects on cognition and mood (Russell et al., 2019; Sharma et al., 2021). Total hemoglobin (Hgb), a protein in red blood cell, ensures adequate tissue oxygenation which is critical to meet physical needs (Otto et al., 2017). Hgb is presumed to decline with aging and has also been associated with cognitive impairment and altered mood due to inadequate delivery of oxygen to the brain (Agrawal et al., 2019). While previous literature has confirmed the interactive relationship between sex hormones, biomarkers, and psychological symptoms (Conde et al., 2021; Davey, 2013), the interactive relationship among these three constructs has not yet been thoroughly examined. In addition, racial/ethnic differences exist in the level of sex hormones, biomarkers, and psychological symptoms (Compernolle et al., 2021; Díaz-Venegas et al., 2016; Kim et al., 2012; Patel et al., 2007). However, the racial/ethnic differences in the interactive relationship among sex hormones, biomarkers, and psychological symptoms remain unknown.

The moderated network model (MNM) may be an intuitive tool to understand and analyze the interactive relationship among sex hormones, biomarkers, and psychological symptoms (Haslbeck et al., 2019). To date, pairwise network model such as the Gaussian Graphical Model (GGM) has been widely used to analyze the dependencies in multivariate data. However, it assumes that each pairwise interaction is independent of all other variables in the network, which does not consider the moderation effects (Haslbeck et al., 2019). The MNM is an extension of GGM with moderation effects, similar to the moderation effects in the linear regression models (Haslbeck et al., 2019). In MNM, the grouping variable is included as the moderator variable, and the group differences are estimated through calculating the moderation effects (Haslbeck, 2022). Herein, network analysis is used to provide a graphic visualization of the MNM where “nodes” represent the variables of interest and “edges” represent the regularized partial correlation between the two nodes after adjusting all other variables within the network (Haslbeck et al., 2019).

In the current study, we aim to use the moderated network analysis and include race as the moderator to examine the moderated effects of race on the interactive relationship among sex hormones (e.g., estradiol, progesterone), biomarkers (e.g., hemoglobin), and psychological symptoms (e.g., cognition, depression, anxiety, loneliness) in female older adults.

## Materials and Methods

### Design and Data Collection

This is a secondary data analysis using the cross-sectional data of Wave II (2010–2011) from the National Social Life, Health, and Aging Project (NSHAP). A total of three waves (Wave I, Wave II, and Wave III) are publicly available, and Wave II was selected for this study due to an adequate sample size and the availability of study variables required to fulfill our study objectives.

### Description of the Data Set

The NSHAP is a longitudinal, population-based study to understand the overall physical, psychological, cognitive, and social health of older, community-dwelling adults in the United States. It collected information on sociodemographic and clinical characteristics, including biomarkers, through the use of study-designed questionnaires and standardized protocols. In addition, biomarkers were collected through a structured process involving both biological samples and comprehensive assessments. Trained interviewers visited participants’ homes to administer health surveys and collect biological samples. Blood samples were drawn using standard venipuncture techniques, allowing for the analysis of various health indicators, including cholesterol levels, total hemoglobin, glucose, and inflammatory markers. Urine samples were also collected to measure additional biomarkers related to metabolic health and kidney function. In some cases, saliva samples were obtained to assess hormonal levels, such as estradiol and progesterone. Each wave was conducted with a 5-year span with Wave I in 2005 to 2006, Wave II in 2010 to 2011, and Wave III in 2015 to 2016. More details about the data set could be found elsewhere (Ho et al., 2018; Olivieri-Mui et al., 2022).

### Participants

Older adults aged between 65 years and 85 years old who reported their gender as female were selected for the current study (Bohannon & Magasi, 2015; Bugos et al., 2007). Those with severely impaired cognition on Montreal Cognitive Assessment (MOCA) (score of <10) were excluded due to their limited ability to complete the study questionnaires. Thus, a total of 1,228 females met the study eligibility criteria.

### Measures

#### Sex Hormones: Estradiol and Progesterone

The salivary enzyme immunoassays were conducted where the participants were asked to submit a sample of 1 mL of unstimulated saliva into a vial via a small household plastic straw. The collected samples were transferred immediately to the Biomeasure Laboratory to minimize bacterial growth and sample degradation (Kozloski et al., 2014). More details on NSHAP’s procedure on collecting hormone assays could be found elsewhere (Gavrilova & Lindau, 2009).

#### Biomarker: Total Hemoglobin (Hgb)

Total hemoglobin (Hgb) was collected from the dried blood spot samples collected using a standardized protocol during the interview. The validity of dried blood spot sample method has shown good sensitivity and precision when compared to venous blood draws (Williams & McDade, 2009). Hgb levels from the dried blood spot samples were converted to whole-blood equivalent Hgb (g/dL) (O’Doherty et al., 2014).

#### Psychological Symptoms: Cognition

The survey-adaptation of Montreal Cognitive Assessment (MoCA), MoCA-SA, consists of 18 questions and was used to measure level of cognition among the older adults. It has shown good internal reliability of Cronbach’s alpha of .76. The total score of MOCA-SA ranges from 0 to 20, and this score could be reliably converted into the original MoCA score that ranges from 0 to 30 using the high-fidelity prediction equation. A higher score on MOCA indicates a better level of cognition (Dale et al., 2018; Kotwal et al., 2016).

#### Psychological Symptoms: Depression

The 11-item Center for Epidemiological Studies Depression (CESD-11) scale was used to measure depression. It has shown good internal reliability with Cronbach’s alpha of .80. The total score of CESD-11 ranges from 0 to 33, with each item asked on a 4-point Likert scale from 0 (“*rarely or none of the time*”) to 3 (“*most of the time*”). A higher score on CESD-11 indicates more severe depressive symptoms (Pun et al., 2017). We regarded a score of 0 to 15 as no or minimal depressive symptoms, 16 to 24 as mild to moderative depressive symptoms, and 25 or higher as severe depressive symptoms (Pun et al., 2017).

#### Psychological Symptoms: Anxiety

The 7-item Hospital Anxiety and Depression Scale (HADS) was used to measure anxiety. The total score ranges from 0 to 21, with each item asked on a 4-point Likert scale from 0 (“*rarely or none of the time*”) to 3 (“*most of the time*”). A higher score on HADS indicates more severe anxiety (Pun et al., 2017). While HADS is commonly used in hospital settings, it has shown well-established reliability and validity in population-based studies (Mykletun et al., 2001).

#### Psychological Symptoms: Loneliness

The 3-item UCLA-loneliness scale was used to measure three dimensions of loneliness, which are relational connectedness, social connectedness, and self-perceived isolation. It has shown good internal reliability with Cronbach’s alpha of .81. The total score ranges from 0 to 6, with each item asked on a 3-point Likert scale from 0 (“*hardly ever*”) to 2 (“*often*”). A higher score on UCLA-loneliness scale indicates more loneliness (Shiovitz-Ezra & Leitsch, 2010).

### Ethical Considerations

The NSHAP study database, codebook, and survey questionnaires are currently publicly available from the Inter-university Consortium for Political and Social Research (ICPSR) website. All de-identified datasets, codebooks, and statistical programs were stored on a secure server at the Columbia University. This study has received an institutional review board declaration of exemption [IRB-AAAU4294].

### Data Analysis

The package “mgm” available in R statistical software program (version 4.0.5) was used to conduct data analysis that allows computation of conditional (in)dependence relationships among mixed types of variables (e.g., continuous, categorical). The moderated MGM was built with race as a categorical moderator (set as “*c*” for “categorical”) and sex hormones, biomarkers, and psychological symptoms as continuous variables (set as “*g*” for “Gaussian”). The visualization of network consists of nodes (variables of interest) and edges (relationship between the nodes), where the width of edges represents the strength of the relationship between variables (Haslbeck, 2022; Haslbeck et al., 2019; Tejada-Gallardo et al., 2022).

The “mgm” employs a regularization parameter with a *L*1-regularized nodewise regression algorithm. Herein, we used the regularization parameter with cross-validation with a hyperparameter γ of 0 and OR-rule to compute pairwise and interaction estimates. Cross-validation is a less conservative model, when compared with other regularization parameters such as the extended Bayesian information criterion (EBIC), but is preferred with small samples due to its high sensitivity, and has been used in previous studies where small sample size is an issue (Tejada-Gallardo et al., 2022). To test the stability of the estimated parameters in the MNMs, we used the *resample ()* function and applied 500 bootstrapped samples. The *plotRes*() function produced a plot of the 500 bootstrapped sampling distribution of the two-way (pairwise) and three-way interaction. This plot shows the moderation effects for each edge with 95% confidence interval, as well as the proportion of nonzero moderated edges across all bootstrap estimations. Nonzero values with 95% confidence intervals indicate the likelihood of moderation effects in the network model (Haslbeck, 2022; Haslbeck et al., 2019; Tejada-Gallardo et al., 2022).

## Result

### Sample Characteristics

Details on participant characteristics are presented in Table 1. The mean age of the White group was 68.91 years (*SD* 6.08) and non-White group was 68.75 years (*SD* 5.73). There was a higher percentage of non-White group who received education less than high school (36.92%) and who were separated, divorced, or widowed (60.28%). The annual household income was lower in the non-White group with more than half with $0 to $24,999 (63.95%) and $25,000 to $49,999 (23.13%). Statistically significant differences were found in the level of education, marital status, annual household income, level of estradiol, total hemoglobin, and cognition between the White and non-White group (*p* < .05).

**Table 1.** Sample Characteristics by Race.

	*n* (%), or mean ± standard deviation		
Variable	White (*n* = 944)	Non-White (*n* = 284)	*p*-value
Age	68.91 ± 6.08	68.75 ± 5.73	.73
Education			<.001***
Less than high school	95 (14.44)	79 (36.92)	
High school graduate or GED	196 (29.79)	49 (22.90)	
Some college or certificate	237 (36.02)	59 (27.57)	
Bachelor’s degree (or more)	130 (19.76)	27 (12.62)	
Marital status			<.001***
Single, never married	17 (2.58)	10 (4.67)	
Married, living with a partner	393 (59.73)	75 (35.05)	
Separated, divorced, widowed	248 (37.69)	129 (60.28)	
Annual household income			<.001***
$0–24,999	143 (32.80)	94 (63.95)	
$25,000–49,999	137 (31.42)	34 (23.13)	
$50,000–99,999	118 (27.06)	14 (9.52)	
$100k or higher	38 (8.72)	5 (3.40)	
Estradiol	3.85 ± 3.89	3.26 ± 3.50	.04*
Progesterone	41.53 ± 88.25	38.75 ± 42.25	.52
Total hemoglobin	12.91 ± 1.94	12.18 ± 2.07	<.001***
Depression	9.97 ± 3.82	10.04 ± 4.08	.77
Anxiety	5.86 ± 3.09	5.67 ± 3.49	.51
Loneliness	1.12 ± 1.45	1.33 ± 1.45	.07
Cognition	24.23 ± 3.32	21.19 ± 4.08	<.001***

*Note*. *p < .05. **p < .01. ***p < .001.

### Moderated Network Model

**Figure 1.**
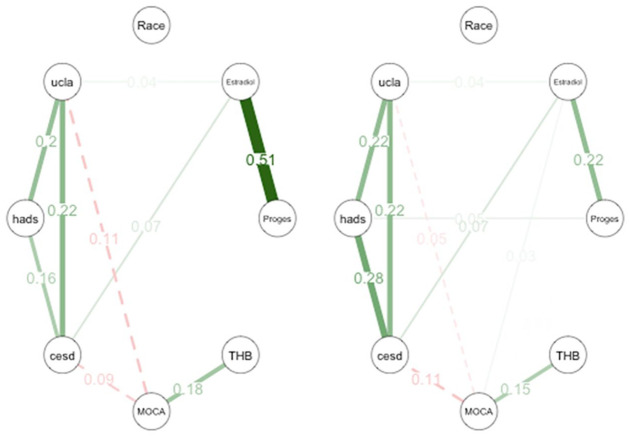
Race, sex hormones/biomarkers, and psychological symptoms *(left: White; right: non-White).* *Note*. UCLA = loneliness; HADS = anxiety; CESD = depression; MOCA = cognition; THB = total hemoglobin; prog = progesterone; estradiol = estradiol.

**Figure 2.**
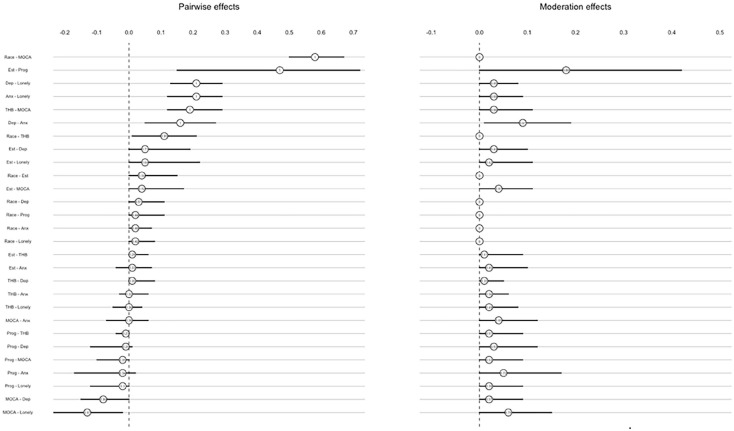
Stability estimates of race, sex hormones/biomarkers, and psychological symptoms. UCLA = loneliness; HADS = anxiety; CESD = depression; MOCA = cognition; THB = total hemoglobin; prog = progesterone; estradiol = estradiol.

When comparing the two groups in the relationship among sex hormones, biomarkers, and psychological symptoms, the White group had a more positive relationship between total hemoglobin and cognition (edge weight = 0.18). Herein, the moderated edge weights can be interpreted as standardized β coefficients. Values closer to 0 suggest a weak relationship, values around 0.5 indicate a strong relationship, and values approaching 1 signify a very strong relationship (Neal, 2024). The moderated edge weight was 0.22, and stability analyses found that this moderated edge weight was nonzero in 66.0% of bootstraps. On the other hand, the non-White group had a positive relationship between progesterone and anxiety (edge weight = 0.05) and between estradiol and cognition (edge weight = 0.03), both of which were not present in the White group. The moderated edge weight between progesterone and anxiety was 0.04, which was nonzero in 72% of bootstraps, and the moderated edge weight between estradiol and cognition was 0.03, which was nonzero in 47% of bootstraps. Figure 1 provides a visualization of the MNM by race (White vs. non-White). Figure 2 depicts the bootstrapped sampling distributions of the MNMs and provides stability estimates.

## Discussion

To the best of our knowledge, this is the first study to use moderated network models to examine the moderated effects of race on interactive relationship among sex hormones, biomarkers, and psychological symptoms in female older adults. Our study found that the degree of some relationships among sex hormones, biomarkers, and psychological symptoms differed by race (White vs. non-White). In addition, a positive relationship between progesterone and anxiety and between estradiol and cognition was identified in the non-White group, both of which were not identified in the White group. Our study highlights the significant influence of social determinants of health on the interactive relationship between sex hormones, biomarkers, and psychological symptoms particularly among the non-White group of female older adults.

A stronger positive relationship between hemoglobin and cognition was identified in the White group. Previous studies have supported the positive relationship between hemoglobin and cognition across different racial groups and reported that cognitive functions are strongly related to level of hemoglobin, which explains why anemic patients experience mild to moderate cognitive decline irrespective of etiology (Agrawal et al., 2019; Qin et al., 2019). In addition, this cognitive decline tends to improve with a higher hemoglobin concentration among middle-aged and older adults (Qin et al., 2019). When comparing between the White and non-White group, the level of hemoglobin tends to be lower in African Americans (Denny et al., 2006). It has been proposed that the difference in level of hemoglobin between the two groups may be due to certain social determinants of health including socioeconomic and nutritional factors (Beutler & West, 2005). For example, low income leads to poor food intake in low nutritional value, which results in iron deficiency anemia in the older, African American adults (Le, 2016). In addition, genetic differences, such as α-thalassemia deletion, may account for the different hemoglobin levels between the Whites and African Americans (Beutler & West, 2005). While anemia is more prevalent in the non-White group (Denny et al., 2006), our study indicates that cognition is more affected by the level of hemoglobin in the White group. Yet, it is important to understand that their difference in the strength of the relationship between the two groups was small (moderated edge weight difference = 0.03). Thus, clinicians should consider that there may be a difference in the effect of hemoglobin concentration on symptoms that presents differently in different racial groups. In addition, clinicians should assess cognition with reference to hemoglobin monitoring in this population.

In contrast, a positive relationship between progesterone and anxiety was identified in the non-White group that was not captured in the White group. Sex hormones play an important role in female physiology and are responsible for maintaining tissue organs, including the central nervous system (Schnall et al., 2019). With aging, female adults experience hormonal changes such as decrease in progesterone level, which tends to be more evident in those undergoing menopause (O’Connor et al., 2009). The fluctuating level or lack of progesterone has been associated with mood disturbances among menopausal women (Joffe et al., 2020). Among female older adults, they receive hormone supplementation therapy with progesterone and other sex hormones that are presumed to improve quality of life and decrease age-related symptoms (Schwartz & Holtorf, 2011). Conversely, the role of progesterone may have an adverse effect on mood in older adults, especially in the presence of stress (Sundström-Poromaa et al., 2020). Progesterone can be converted into cortisol, which is a stress hormone, and leads to stress response associated with mood alteration and impaired emotional processing (Sundström-Poromaa et al., 2020). Among the non-White female older adults, exposure to cumulative experiences of racism often results in limited access to resources, exacerbating health disparities and increased mental health disorders (Goosby et al., 2018). As a result, clinicians should carefully consider the risks and benefits of recommending hormone supplementation therapy especially to non-White female older adults, where progesterone can cause higher level of anxiety.

Furthermore, we found a positive relationship between estradiol and cognition in the non-White group. Previous research has shown that estradiol has salutatory neurophysiologic effects and modulates cognitive function (Im et al., 2019; Luine, 2014; Sundström-Poromaa et al., 2020; Wise et al., 2001). The level of circulating estradiol decreases with aging which leads to age-related declines in learning and memory function (Luine, 2014). In a randomized study, postmenopausal women received a high dose of estradiol by skin patch for 8 weeks and significant effects on attention, verbal memory, and visual memory were reported (Asthana et al., 2001). Compared to midlife women, the degree of hormonal changes may be subtle at older stage of life (Schwartz & Holtorf, 2011). However, older adults are more likely to receive hormone supplementation therapy, which may lead to changes in their sex hormone levels and affect cognition (Schwartz & Holtorf, 2011). As estradiol may improve cognition among older adults, clinicians should continuously monitor the level of estradiol and consider hormone mentation therapy, especially for non-White female older adults.

There are several limitations to the study. First, we used the regularization parameter with cross-validation and the OR-rule to compute pairwise and interaction estimates. While cross-validation is recommended for small sample size, this parameter is a less conservative model, and may result in false positive results. Thus, future studies should use more conservative regularization parameters such as the EBIC with a bigger sample size to obtain more reliable results. Second, the sample size was greater for the White group than the non-White group even though the NSHAP oversampled African American adults in the second wave. Thus, the moderation effect identified was small. Future studies need to be conducted with a larger sample size of more diverse racial groups to detect a greater moderation effect. Third, different measurement standards (objective vs. subjective) were used to measure sex hormones, biomarkers, and psychological symptoms. As a result, there is potential for downward bias due to the differences in their measurement domains. Fourth, we used cross-sectional data to understand the moderation effects of race on the relationship among sex hormones, biomarkers, and psychological symptoms. As the level of sex hormones, biomarkers, and psychological symptoms might change over time, longitudinal approach (e.g., longitudinal network analysis) is needed to understand the temporal changes in their relationship.

## Conclusion

The current study examined the differences in the relationship among sex hormones, biomarkers, and psychological symptoms by race, and how this relationship is further moderated by race using moderated network model. The type and strength of relationship among sex hormones, biomarkers, and psychological symptoms were different between the White and non-White group. In addition, a small moderated effect of race was identified. Despite the study limitations, our study offers important preliminary findings to understand the potential racial disparities with an interactive approach among female older adults.
